# Targeted endomyocardial biopsy guided by real-time cardiovascular magnetic resonance

**DOI:** 10.1186/s12968-017-0357-3

**Published:** 2017-04-19

**Authors:** Christina Unterberg-Buchwald, Christian Oliver Ritter, Verena Reupke, Robin Niklas Wilke, Christine Stadelmann, Michael Steinmetz, Andreas Schuster, Gerd Hasenfuß, Joachim Lotz, Martin Uecker

**Affiliations:** 1 University Medical Center Goettingen, Clinic of Cardiology and Pneumology, Goettingen, Germany; 2 Department of Experimental Animal Medicine, Georg-August University, Goettingen, Germany; 3 DZHK (German Centre for Cardiovascular Research), Partner Site Goettingen, Berlin, Germany; 4 Department of Neuropathology, University Medical Center Goettingen, Goettingen, Germany; 5 University Medical Center Goettingen, Clinic of Pediatric Cardiology and Intensive Care Medicine, Goettingen, Germany; 6 University Medical Center Goettingen, Institute for Diagnostic and Interventional Radiology, Goettingen, Germany

**Keywords:** Endomyocardial biopsy, CMR, Targeted biopsy, Real-time MRI

## Abstract

**Background:**

Endomyocardial biopsies (EMB) are an important diagnostic tool for myocarditis and other infiltrative cardiac diseases. Routinely, biopsies are obtained under fluoroscopic guidance with a substantial radiation burden. Despite procedural success, there is a large sampling error caused by missing the affected myocardium. Therefore, multiple (>6) biopsies are taken in the clinical setting. In cardiovascular magnetic resonance (CMR), late gadolinium enhancement (LGE) depicts areas of affected myocardium in myocarditis or in other infiltrative cardiomyopathies. Thus, targeted biopsy under real-time CMR image guidance might reduce the problem of sampling error.

**Methods:**

Seven minipigs of the Goettingen strain underwent radiofrequency ablation in the left ventricle. At least two focal lesions were induced on the lateral wall in five and the apex in two animals. Each ablation lesion was created by two consecutive 30 sec ablations (max. 30 W, temperature 60–64 °C). Biopsies were taken immediately after lesion induction using a commercially available 7 F conventional bioptome under fluoroscopic guidance at the ablation site. Afterwards the animals underwent CMR and lesion visualization by LGE at 3T. The lesions were then targeted and biopsied under CMR-guidance using a MR-conditional bioptome guided by a steerable catheter. Interactive real-time (RT) visualization of the intervention on an in-room monitor was based on radial FLASH with nonlinear inverse reconstruction (NLINV) at a temporal resolution of 42 ms. All samples underwent a standard histological evaluation.

**Results:**

Radiofrequency ablation was successful in all animals. Fluoroscopy-guided biopsies were performed with a success rate of 6/6 minipigs - resulting in a nonlethal pericardial effusion in one animal. Visualization of radiofrequency lesions by CMR was successful in 7/7 minipig, i.e. at least one lesion was clearly visible. Localization and tracking of the catheters and the bioptome using interactive control of the imaging plane was achieved in 6/6 MP; however in the animal with a large pericardial effusion after EMB under fluoroscopy no further EMB was attempted for safety reasons. Biopsies under interactive RT-CMR guidance were successfully performed in 5/6 animals, in one animal the bioptome reached the lesion, however the forceps did not cut out a sample. Specimens obtained under CMR guidance contained part of the lesion in 6/15 (40%) myocardial specimens and in 4/5 (80%) animals in which samples were achieved. Conventional biopsies revealed ablation lesions in 4/17 (23.5%) specimens in 3/6 minipigs (50%).

**Conclusion:**

Focal lesions induced by radiofrequency ablation in a minipig model are a useful tool for CMR-guided biopsy studies. In contrast to fluoroscopy, CMR provides excellent visualization of lesions. Interactive real-time CMR allows excellent passive tracking of the instruments and EMB provides significantly superior sampling accuracy compared to fluoroscopy-guided biopsies. Nonetheless, further improvements of MR-compatible bioptomes and guiding catheters are essential before applying this method in a clinical setting.

**Electronic supplementary material:**

The online version of this article (doi:10.1186/s12968-017-0357-3) contains supplementary material, which is available to authorized users.

## Background

Endomyocardial biopsies (EMB) are an important diagnostic tool in myocarditis, arrhythmias, cardiac tumors, storage disease, cardiac allograft rejection and other cardiac diseases with unknown origin. With DNA and RNA detection EMB evolved into an important diagnostic tool [1, 2] proposed by the AHA and ACC [3] as well as ESC [4]. However, there are concerns about the large sampling error and the adequate clinical setting for biopsies is still in debate. Therefore, in today’s clinical routine multiple (5–6) biopsies are taken from either the left or right ventricle under fluoroscopic guidance with a radiation burden for the patient and the interventionalist. Despite procedural success with overall (major and minor) complication rates from 0% [5] to 5% [6, 7] missing the affected myocardium results in a limited diagnostic value providing a diagnostic result in only 25.5% of clinical cases [6]. Thus, it seems desirable to use a method for visualization of the diseased parts of the heart in order to take directed samples from the affected myocardium. Cardiovascular magnetic resonance (CMR) has a superior soft-tissue contrast compared to X-ray [8, 9] and allows arbitrary orientation of the imaging plane in three dimensions without exposure to ionizing radiation. Hence, targeted EMB under real-time CMR guidance could solve the sampling problem by reducing the need for multiple biopsies. Nevertheless, interventional CMR has to solve several technical challenges. First, MR-safe and suitable guidewires as well as steerable guiding catheters with distal-tip visualization are mandatory to navigate and reliably reach the affected myocardium. Furthermore, the magnetic field does not allow the use of conventional metallic bioptomes due to heating, magnetization and massive metal artifacts. Until now, MR biopsies are mostly applied in non moving organs like breast [10], liver [11], kidney [12], prostate [13] or brain [14] using MR-compatible needles. MR-safe cardiac bioptomes are still not commonly available. Lossnitzer [15] evaluated a preclinical MR-conditional bioptome in an ex vivo animal heart model. The NIH group [16] recently demonstrated the feasibility of CMR-guided EMB in an in vivo swine model with extended infarct scars. However, clinically many cardiac conditions are associated with small circumscript lesions rendering targeted biopsies even more challenging. Therefore, we developed an animal model with distinct left ventricular lesions created by controlled radiofrequency ablation. The aim of our study was to show that targeted EMB of focal myocardial lesions is feasible by real-time CMR guidance using a clinical 3T scanner, i.e. that continuous real-time CMR in three dimensions enables a controlled positioning of the guidewire, the guiding catheter, the bioptome and the biopsy itself. Furthermore, we aimed at showing that targeted biopsy has a lower sampling error under real-time CMR compared to fluoroscopically controlled biopsies.

## Methods

### Animal model

Seven minipigs of the Goettingen strain weighing 30–46 kg and aged 12–24 months received diazepam at a dosage of 0.5 mg/kg oral and 2 mg/kg azaperone and 10 mg/kg ketamine i.m. as premedication. Anesthesia was induced by 40–60 mg of propofol and 100–150 μg of fentanyl. Anesthesia was maintained by 3–4 Vol% of sevoflurane in 50% oxygen and 50% air and 5–10 μg/kg/h fentanyl. Postoperative analgesia was provided by 50 mg/kg metamizole i.v. and 2.2 mg/kg flunixin i.m.. Blood gases were regularly monitored and ventilation was adjusted to maintain blood gases in the physiologic range. Surface electrocardiogram, concentration of carbon dioxide and oxygen level in the blood were monitored using MR-compatible LCD monitoring system (Precess 3160, Invivo, Orlando, Florida, USA).

All experiments consisted of five parts:

1. radiofrequency ablation in the left ventricle for creation of circumscript lesions under fluoroscopic guidance,
2. endomyocardial biopsy (aim: 3 specimens) under fluoroscopic guidance,
3. standardized CMR for characterization and lesion localization,
4. targeted endomyocardial biopsy using real-time MRI (aim: 3 specimens),
5. histology of the obtained endomyocardial biopsies.

**Fig. 1:**
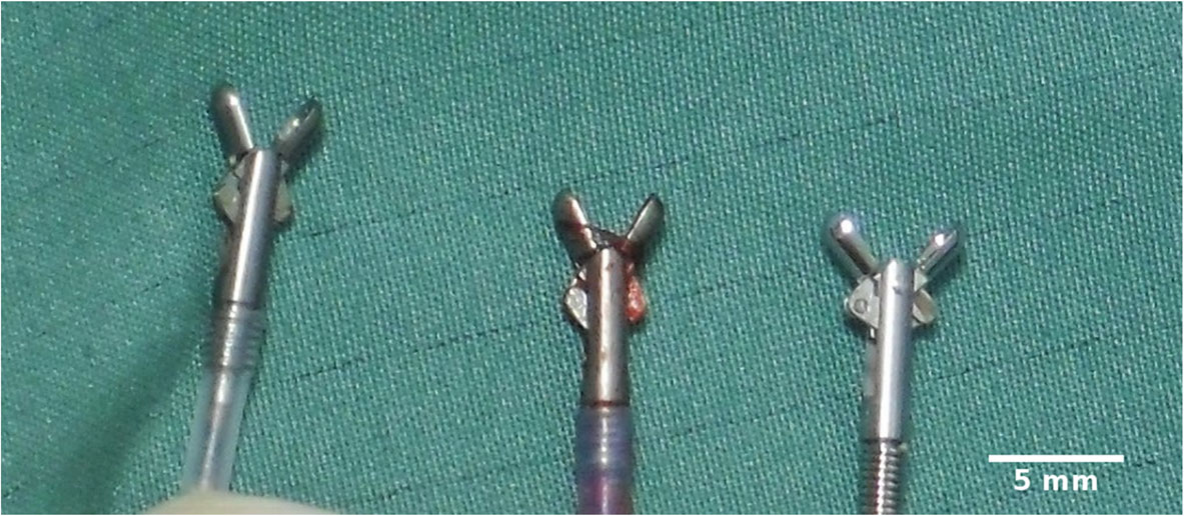
Bioptomes. Bioptomes with forceps in the open state: the two bioptomes on the left are MR-conditional, the one on the right hand is a standard steel bioptome employed for EMB under fluoroscopic guidance. Notably, the standard bioptome achieves a maximum opening angle of about 90° whereas the MR-bioptomes only open at a maximum angle of about 50–70°

A femoral artery (*n* = 4) or a carotid artery (*n* = 3) was punctured and a 10 French (F) introducer sheath was placed. A standard 7 F (in one case 5 F) deflectable ablation catheter (4-mm electrode without cooling, Marinr™, Medtronic, USA) was advanced into the left ventricle under fluoroscopic guidance. The intracardiac electrogram (ECG) was recorded by a dedicated electrophysiology system (Prucka, GE Healthcare, USA). Radiofrequency lesions were created on the endocardial surface with a clinical-grade radiofrequency generator (HAT 300 S, Osypka, Germany) using a power-controlled mode at 30 W for 30 s (once) or 2x30s at all other ablation sites. The ablation catheter was positioned to the lateral wall. Position was controlled via angulation of the c-arm x-ray in 30^o^ RAO and 45^o^ LAO (Siremobil, Siemens Healthineers, Erlangen, Germany). The intracardiac electrogram (bipolar with sharp ventricular potential and no atrial electrogram) was monitored. No other cardiac imaging – either echocardiogram, cardiac ventriculography or computer tomography - was performed prior to radiofrequency ablation and X-ray guided endomyocardial biopsy. At least 15 min. post ablation an 8.5 F deflectable guiding catheter was introduced into the left ventricle. The guiding catheter (St.Jude Medical, St. Paul, USA, Fast-Cath, SRO, 8.5 F) was advanced to the ablated myocardium under fluoroscopic guidance using the same angulations of the radiofrequency procedure with help of the stored pictures of the radiofrequency ablation sites as guidance. Endomyocardial biopsies were then taken utilizing a conventional 5.5 F cardiac bioptome (biopsy forceps Cordis, Cardinal Health, Dublin, Ireland; volume of samples approx. 2.46 mm^3^) (Fig. 1). Time from introduction to extraction of the guiding catheter varied between 15–25 min for all biopsies. Ablations and biopsies under X-ray and under CMR were performed by one cardiologist with more than 20 years’ experience in cardiac interventions (radiofrequency-ablations, angioplasties and biopsies) with the help of an interventional radiologist (>15 years’ experience in interventions).

### CMR

The animal was then placed into a 3T clinical MR scanner (Skyra, Siemens Healthineers, Erlangen, Germany). A cardiac 18-channel body array receive coil (Siemens Healthineers, Erlangen, Germany) was positioned on the chest. After standard T1-weighted scout images, images for function were obtained with standard bSSFP sequences in standard views (three long axis views and a stack of short axis views) followed by real-time movies using radial bSSFP with NLINV reconstruction (temporal resolution 33 ms; spatial resolution: 1.6 mm × 1.6 mm; slice thickness: 6 mm; FOV: 256 mm × 256 mm; matrix size: 160 × 160; TR = 2.56 ms; TE = 1.28 ms; flip angle: 26^0^; bandwidth 1270 HZ/pixel; 13 projections per frame) [17]. For LGE 1 mmol/kg body weight gadobutrol (Gd-BT-DO3A, Gadovist®, Bayer Schering Pharma AG, Leverkusen, Germany) was injected intravenously. Scars were depicted 15 mins after contrast injection using a standardized inversion recovery turboFLASH technique (spatial resolution 1.4 mm × 1.4 mm; slice thickness: 4 mm; FOV: 360 mm × 360 mm; matrix size: 256 × 256; retrospective gating; TR = 919 ms; TE = 1.41 ms, flip angle: 40^0^; bandwidth 780 Hz/pixel).

An 8.5 F deflectable guiding catheter (Innovative Tomography Products, Bochum, Germany) with a 0.035 inch MR-conditional guidewire (MaRVis Medical, Hannover, Germany) was introduced into the left ventricle by a retrograde approach. The wire consists of glass and aramid fibers as well as epoxy resin. Metal particles are embedded in an envelope polymer and covered by a polytetrafluoroethylene shrink tube as the outer surface (information is provided on the website of the manufacturer [18]). The guiding catheter is comparable to a standard deflectable catheter but the braiding is replaced by special nonmetallic fibers. These are also part of the traction element. The distal tip is visible due to a very small ring made of nonmagnetic steel. This allows visualization under MRI as well as under X-ray. The artifacts under CMR are very small (see Additional file 1: Video). A multi-GPU computing system (BiomedNMR, Göttingen, Germany) designed for low-latency online image reconstruction was used with radial FLASH sequences for interactive real-time MRI [19]. This system allowed for immediate image display of real-time images and fast interactive sequence control. Here, a radial FLASH sequence with NLINV reconstruction optimized for interactive real-time MRI was used (temporal resolution: 42 ms; spatial resolution: 2 mm × 2 mm; slice thickness: 8 mm; FOV: 256 mm × 256 mm; matrix size: 128 × 128; TR = 2.02 ms, TE = 1.3 ms, flip angle: 8°, bandwidth: 1700 Hz/pixel, 21 projections per frame) [19]. In particular, images could be shown on the operating console of and on a MR-compatible in-room monitor (NordicNeuroLab, Bergen, Norway) with a time-delay of about 0.27 s providing an excellent visual feedback for the interventionalist. The time-delay includes the time for acquisition, data transfer, image reconstruction, post-processing and image display. It was measured by simultaneously tracking the motion of a moving water phantom using the real-time CMR sequence with the same parameters as during intervention and visible laser light. During an intervention, procedural adjustments of the image planes were performed by a trained technician at the console outside the scanner room cage based on communication with the interventionalist inside using the Imroc IR™ communication system (Optoacoustics, Mazov, Israel). Lesion size and location were clearly depicted by phase sensitive reconstructed turboFLASH inversion recovery late gadolinium enhancement imaging (PSIR-LGE). Images in three different planes were stored on the in-room monitor and were compared to the images simultaneously acquired by real time imaging. The MR-conditional bioptome (Innovative Tomography Products, Bochum, Germany) (Fig. 1) was introduced and guided towards the lesion under continuous visual control and interactive adjustment of the imaging plane. Papillary muscles, aortic valve as well as mitral valve served as landmarks. The final position of the bioptome was adjusted step by step: the tip of the device was kept in plane and directed to the target area that was visible on stored LGE images on the in-room monitor. The most difficult part of the procedure was the positioning of the deflectable guiding catheter due to its rigidity and diameter. The MR-conditional guiding catheter had more rigidity and less flexibility than the guiding catheter used under fluoroscopy.


Additional file 1: Real-time CMR during endomyocardial biopsy. (MPG 2996 kb)


Overall tracking was acquired with passive tracking via visible markers on the guidewire, on the distal tip of the guiding catheter (Innovative Tomography Products, Bochum, Germany) and via distal markers of the bioptome (Innovative Tomography Products, Bochum, Germany).

For later quantification of LGE a semiautomatic gray-scale threshold technique (Qmass, Medis, Leiden, The Netherlands) was performed as published previously [20]. Areas of LGE were defined as a signal intensity of more than +4 standard deviations (SD) above the mean of remote healthy myocardium.

### Histology

All samples were retained in a 10% buffered formalin solution and remained there for at least 24 h. Afterwards they were further processed including paraffin embedding and sectioning. Sections of 5 μm thickness were stained using hematoxylin-eosin, periodic acid-Schiff, elastic van Gieson staining as well as desmin immunohistochemistry to identify healthy and damaged myocardium, endocardium and hemorrhage. Blinded histologic evaluation was performed by two experienced physicians. Myocardial interstitial and intracellular edema and vacuolization, myocardial pallor, myocardial coagulation and hemorrhage were assessed and reported for each biopsy sample.

All animal protocols were reviewed and approved by the local animal ethics committee as well as the governmental animal care and use committee (Bezirksregierung Braunschweig, Germany).

## Results

### Radiofrequency ablation

Radiofrequency ablation was successful in all 7 MP and performed on the left ventricular lateral wall in five and the apex in two animals. Each (except one) ablation lesion (7-F catheter) was created by two consecutive 30-s ablations (max. 30 W, temperature 58-64 C^0^). Ventricular fibrillation occurred twice in one animal (one minute after the first and 75 s after the second ablation that was limited to 30 s). Successful defibrillation resulted in sinus rhythm followed by stable hemodynamics. At least two ablation sites with sufficient endocardial contact (temperature continuously above 58 C^0^) were achieved in all animals.

### Endomyocardial biopsies

#### Fluoroscopic guidance

The conventional bioptome (Fig. 1) was guided under fluoroscopy (Siremobil, Siemens Healthcare, Erlangen, Germany) as in common clinical routine. Guiding towards the region of interest was accomplished by comparing the actual fluoroscopic image on the screen with the stored images of the ablation procedure using the same angulations of the c-arm x-ray. At least one sample was taken on what was judged to be within the ablation zone. A commercially available steel bioptome (5.5 F) was used in 6/7 animals. Time from introduction to extraction of the guiding catheter varied between 15–25 min for all biopsies. Further complications occurred in two animals: both suffered from pericardial effusion without major hemodynamic problems. Thus, samples were obtained from six animals (Table 1).

**Table 1:** Numbers and pathologic findings in targeted endomyocardial biopsies gained under RT-CMR or fluoroscopic guidance (Fl)

Ablation sites	No. of EMB RT-CMR/No. of trials	No. of EMB Fl/No. of trials	No. of positive samples		Pathology correctly diagnosed/minipig		Lesion size	
			RT-CMR	Fl	RT -CMR	Fl	[g]	[% total LV mass]
3	3/6	0/0	3	0	yes	no	3.1	8.5
2	4/4	4/4	1	0	yes	no	12.8	22.8
3	2/6	1/1	1	0	yes	no	6.0	11
2	2/6	3/3	0	1	no	yes	11.4	20.6
3	0/5	2/2	0	0	no	no	3.5	7.7
3	4/6	3/3	2	1	yes	yes	2.7	5.0
3	0/0	4/4	0	2	no	yes	Not done	

Number (No) of tissue samples obtained by endomyocardial biopsy (EMB) under real-time CMR and under fluoroscopy (Fl), number of trials of biopsies and lesion size in g and in % of total left ventricular (LV) mass are given. No EMB trials were done in the first animal under FL and in the last animal due to large pericardial effusion

#### CMR guidance

**Fig. 2:**
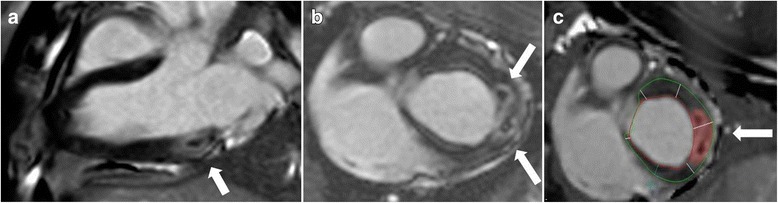
**a**: Postcontrast visualization and assessment of acute lesions. Postcontrast two-dimensional PSIR-LGE (2D LGE) in two different animals after radiofrequency ablation. LGE images show good contrast between the lesion with its edematous core and the myocardium (white arrow). **a**: lesions targeted for biopsy are shown in an infero-basal segment in a long axis view and **b**: in a short axis view with **c**: the markers for quantitative segmentation of the lesion area (red area, see methods)

7/7 animals survived and were transferred into the CMR suite. Detection of the lesion was achieved 100–150 min after ablation. Pre-contrast the lesions were invisible with the used sequences. For LGE, time of inversion (TI) value was between 250 and 300 ms. Images were acquired 15 + 5 min after the body-weight adapted injection of gadubutrol. PSIR-LGE images provided good depiction of the lesions (Fig. 2). As the tip diameter of the ablation catheter was 4 mm, the induced lesions were rather small. As CMR imaging followed standard biopsies in all experiments we firstly ruled out that the biopsy itself led to a CMR detectable lesion in an additional animal. Left ventricular myocardium was analyzed with dedicated CMR software (Qmass, Medis, Leiden, The Netherlands). Lesion size defined as above varied between 3.8% (2.4 g) and 22.8% (12.8 g) of total myocardial mass (0.27 cm^2^ and 4.64 cm^2^ in the best available view), most probably depending on the contact and heating during the ablation. Lesions are given in table 1. Five lesions had a typical appearance with a contact point at the lesion core and an outer rim as described by Celik [21].

Localization and tracking of the guiding catheter with a distal tip marker (ITP, Bochum, Germany) (see Additional file 2) and an MR-safe 0.035 in. guidewire (MaRVis Technologies GmbH, Aachen, Germany) with passive tracking was successful in all animals. The guidewire was visible over the whole length with a distal extra ball-shaped tip marker increasing tip visibility.

**Fig. 3:**
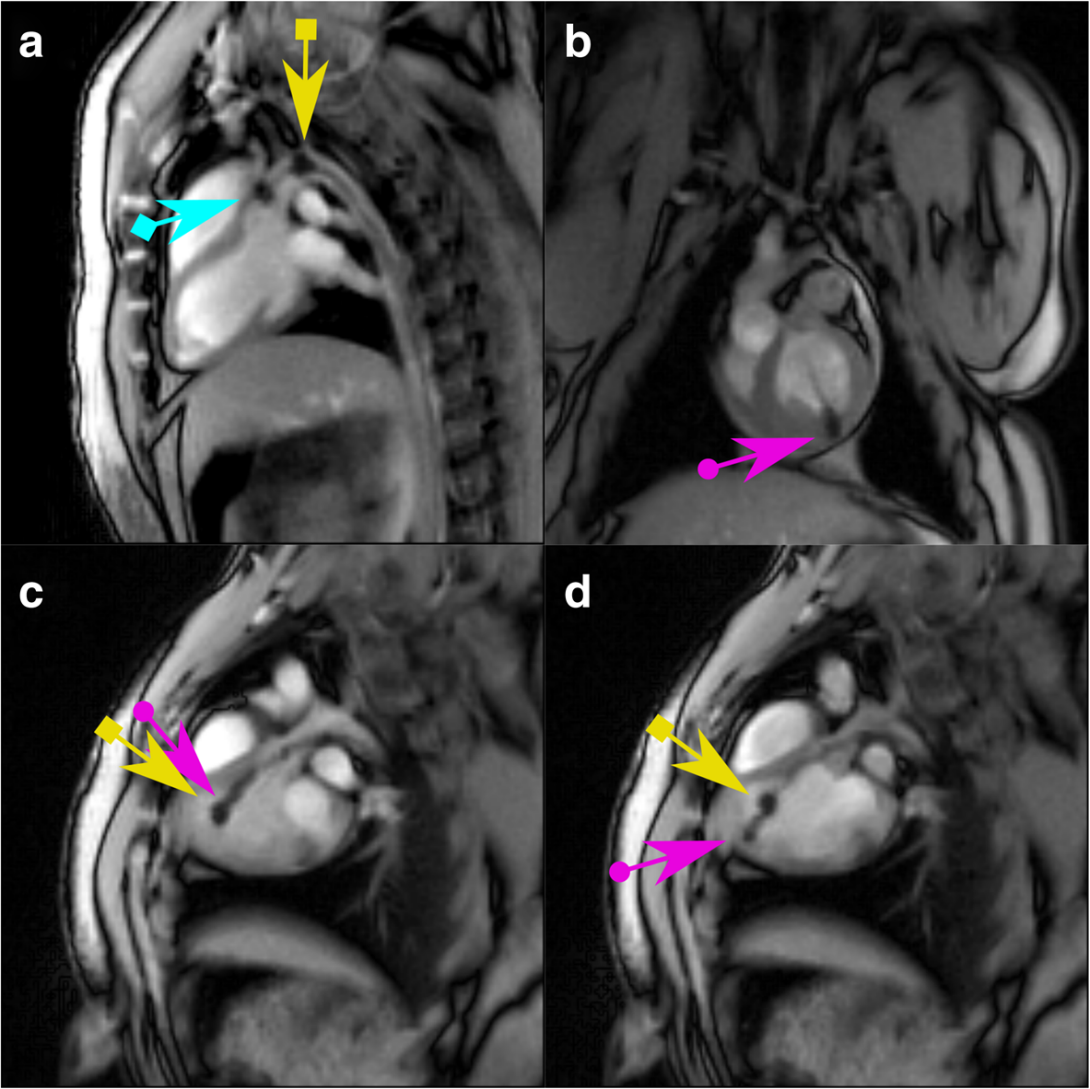
Guidewire, guiding catheter and MR-bioptome visualized by real-time CMR pre biopsy. The instruments with MR-markers are visualized by interactive real-time CMR during a targeted biopsy. **a**: The guidewire (blue arrow with square end) and the guiding catheter (yellow arrow with square end) are advanced into the left ventricle through the aorta. **b**: The MR-conditional bioptome is used to perform a biopsy (violet arrow with round end) **c** and **d**: The bioptome (violet arrow with round end) is advanced inside (**c**) and then extruded out of (**d**) the guiding catheter (yellow arrow with square end)

Due to the availability of in-room real-time monitoring (as described above), the whole interventional procedure was trackable in all animals: advancing the guidewire into the aorta and the left ventricle followed by introduction of the guiding catheter could be safely performed using interactive navigation in different planes (Fig. 3). The MRbioptome’s distal tip was clearly visible and the negative contrast (artifact) allowed simultaneous detection of the endocardial border (see Additional file 2). The most difficult part of the procedure was the positioning of the deflectable guiding catheter due to its rigidity and diameter. The MR-conditional guiding catheter had more rigidity and less flexibility than the guiding catheter used under fluoroscopy. Procedure time for real-time CMR guidance varied between 40–50 min (first introduction of the guiding catheter into the sheeth until extraction after the last biopsy) depending on the difficulty to place the bioptome. In 5/7 animals biopsies were successfully performed: in one minipig the bioptome reached the lesion; however the forceps didn’t cut a sample, in one other MP pericardial effusion was major. In this animal the EMB under CMR guidance was skipped due to safety reasons. Handling of the catheters was tested by a carotid approach (*n* = 3) as well as by a femoral approach (*n* = 4). Continuous navigation was dependent on an optimal communication with the technician outside the scanner room to rotate image planes in the necessary position in a minimum of time. An additional movie file shows this in more detail (see Additional file 2). The handling from the carotid approach was uncomfortable for the interventionalist as geometric constraints required the interventionalist to move his or her head several times in and out of the scanner at the end of the bore which led to headache and slight nausea due to exposure to intense gradient fields during scans. On the other hand, the femoral approach allows for a position where the interventionalist can handle the catheter without having the head in the tunnel.

#### Histology of the myocardial specimen

**Fig. 4:**
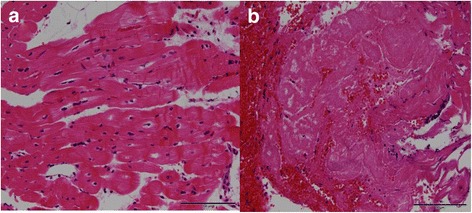
Histology of endomyocardial biopsies. HE staining **a**: The endomyocardial biopsy shows regular myocardium. This specimen was gained under fluoroscopic guidance in minipig 1 (see Table 1). **b**: One of the two specimens obtained under CMR guidance. The ablated area reveals areas of myocardial coagulation with extensive hemorrhage

Samples obtained under CMR guidance included ablation lesions in 6/15 (40%) samples and in 4/5 animals in which biopsies could be performed (Table 1) successfully. EMB obtained under fluoroscopy guidance revealed ablation lesions in 4/17 (23.5%) specimen and in 3/6 (50%) animals in hematoxylin-eosin stain (Fig. 4) or elastic van Gieson stain. Thus, biopsy of lesioned cardiac tissue was achieved with CMR-guided successful biopsies in 4/5 animals and in 3/6 animals taken under fluoroscopic guidance.

## Discussion

Our work is a proof of concept for targeted EMB under real-time CMR guidance in a 3T environment. In a minipig model localized lesions were induced by radiofrequency ablation and visualized using CMR. In our study these lesions served as a useful tool for CMR-guided biopsy studies. We were able to show that biopsies of these lesions can be achieved under CMR guidance with a higher diagnostic yield compared to conventional fluoroscopic guidance in the same animals. We have used this dedicated animal model of defined circumscript lesions to mimic the clinical situation of unknown heart failure in which 6–8 specimens are taken for diagnostic purposes. The Lederman group [16] recently published targeted EMB using CMR guidance in an animal model with significantly larger infarct scars. Their results show a very good correlation to our observations: CMR guidance was superior to fluoroscopic guidance (82% vs 56%) in targeted EMB. Similar to our experience, specimens collected by the MR-conditional bioptome were smaller than those by the conventional steel bioptome. Moreover, we observed that success rates depend on the cutting force of the bioptome. However, this force was markedly less in the MR- bioptome compared to the steel bioptome. The use of the MR-bioptome resulted in a procedural success rate of 45% whereas conventional biopsies showed a rate of 100%. This illustrates a technical problem to be resolved in the future: the sharpness of the forceps was not optimal and inferior compared to a conventional steel bioptome with long metallic compounds and powerful traction of the forceps. The polymer design is optimal for elimination of heating problems but its mechanical rigidity still is not strong enough to transfer enough force to the forceps. Moreover, increasing the length from 80 cm (carotid access) to more than 150 cm (femoral access) resulted in decreased force on the forceps (personal communication of the manufacturer). Our aim was to cut at least three specimens in each setting but this was not achievable in all animals. For the steel bioptome we were successful in all cases, but we had two major complications in the minipigs: one animal suffered from small, hemodynamically non-fatal pericardial effusion and the other one from ventricular fibrillation. This complication rate was considerably higher than reported for left ventricular biopsies in clinical routine which may be explained by the combination of RF ablation immediately followed by EMB. Further, this complication might be the result of using monoplanar instead of biplanar fluoroscopy. Schäufele [5] reported on procedural success rates of 98% and a very low complication rate (0%) for left ventricular biopsies when using modern fluoroscopy and transradial equipment in humans in an elective clinical setting. In a larger clinical series of patients with unexplained heart failure left and right ventricular biopsies were associated with a complication rate of approx. 1.9% [6].

As LGE imaging provides a high contrast between healthy and altered myocardium we used it for visualization and targeting of the lesions before EMB in the CMR scanner. All lesions created with a 7 F ablation catheter were visible. Celik [21] suggested that native T1 contrast may be even more precise in separating the necrotic core from the surrounding edematous rims without waiting for the equilibrium after gadolinium injection. This specific aspect is of high interest for further studies using lesions induced by radiofrequency ablation as this can yield ablation zones early and without recurrent injection of gadolinium. It is suggested that this might be even more useful for localization of the center of the EMB target.

In our study, real-time CMR was applied for navigation of MR-compatible guidewires and catheters in a comparable manner to the procedure under fluoroscopy which is familiar to the interventionalists. In all cases the guiding catheters could be placed into the left ventricle under continuous real-time CMR guidance. Guidewires had an excellent visibility but their polymer-only construction results in a flexibility that was sufficient for the described purpose but not quite the same as that of metallic wires. Torquability and support were not measured and catheter exchange over the wire was not performed. However, the overall handling is inferior to wires that are routinely used in cardiac interventions. Wire shortcomings might be better solved by a segmented nitinol design as invented by the NIH group [22] but these wires are currently not available on the market. Although the use of commercially available nitinol wires is possible in the magnetic field without artifacts, these wires are not safe concerning heating and induction. By use of a prototype MR-guiding catheter the MR- bioptome could be introduced easily into the left ventricle in all cases. Again this is in good agreement with the study of the NIH group [16]. The size and intensity of the artifacts was unproblematic. However, exact placement towards the lesions was more challenging. Consequently required procedure time was more than doubled in the CMR scanner.

Most interventional MRI studies are currently performed using MRI at 1.5 T which has the general advantage of being less prone to artifacts and heating, and because equipment using active tracking is often only developed for this field strength. In our study, we use passive tracking using a real-time imaging method based on radial FLASH and highly accelerated by advanced parallel imaging (NLINV). This method is robust against field inhomogeneities (short TE), is not problematic with respect to heating (no wires and use of low-flip angles), and benefits from the higher SNR and improved parallel imaging at 3T. One major aspect of our study is the use of real-time CMR in a manner comparable to fluoroscopic guidance in a 1:1 imaging setting. Although the used real-time FLASH sequences with NLINV reconstruction allow a comparably high temporal resolution of 20 ms per frame [17], we have reduced the temporal resolution to 42 ms per frame in order to reduce computational load. This guaranteed excellent visual feedback for the interventionalist with a minimal time delay. We were also able to switch between different orientations within a few seconds. This makes the procedure less dependent on pre-scanned images or pre-defined imaging planes.

### Limitations

Heating of the bioptome has not been assessed during imaging in the 3T environment. However, as the forceps are made of titanium and the shaft is made of plastic with aramid fibers these different components should have no major heating problems. In phantom dental implants made of titanium there is an increase of 0.6–0.8 °C in a 3T scanner [23]. As circulating blood has a cooling effect this heating should be no relevant problem. Safety studies will be required prior to clinical translation in regard to the problem of flex shaft, cutting jaw, safety and effectiveness of the MR-conditional bioptome. Compared to a 1.5 T scanner the 3T scanner offers better contrast of the myocardium with improved depiction of acute and chronic lesions but most MR-compatible catheters are approved for 1.5 T only. The mechanical features of MR-compatible guiding catheters such as flexibility, steerability, available different curvature and different sizes are problems still unresolved. We used a non CE approved flexible and deflectable guiding catheter in this work. Until now there is no adequate nonbraided femoral catheter on the market with deflectable tip and with a smaller outer diameter: in our case 8.0 F would have been wide enough, instead we had to use a 9.5 F guiding catheter that is more harmful for all structures. Nevertheless, our techniques should be translatable into clinical and commercially available products. Further improvements in real-time imaging will make the procedure more convenient, i.e. interactive control directly from the scanner room, interactive control of sequence parameters which control the contrast (e.g. saturation pulses), improved user-interface for interventional use with simultaneous 3D visualization of several imaging planes and an overlay of static images will facilitate CMR interventions. Finally, we cannot fully exclude that the pathologic assessment of endomyocardial biopsies may be influenced by the later sampling time point (approx. 120 min) of real-time CMR–guided biopsies.

## Conclusion

Our work shows that focal myocardial lesions can be induced by radiofrequency ablation and afterwards visualized under CMR in a minipig model. These focal lesions can be successfully biopsied. Real-time CMR guidance show a higher rate of diagnostic success compared to biopsies obtained under fluoroscopic guidance. Visualization and passive tracking of the instruments using real-time CMR with interactive control is excellent. Further improvements of bioptomes and guiding catheters will contribute to the superior success of targeted CMR-guided biopsy compared to untargeted fluoroscopic EMB.

## Additional files

Additional file 2: Real-time CMR images seen by the interventionalist during biopsy of a radiofrequency induced lesion similar to the one depicted in Fig. 2. (PPTX 9082 kb)

## Abbreviations

ACC: American College of Cardiology
AHA: American Heart Association
bSSFP: Balanced steady state free precession
BW: Body weight
CMR: Cardiovascular magnetic resonance
DZHK: German Center for Cardiovascular Research (Deutsches Zentrum für Herz-Kreislauf-Forschung)
EMB: Endomyocardial biopsies
ESC: European Society of Cardiology
EVG: Elastic van Gieson
FLASH: Fast low-angle shot
HE: Hematoxylin-eosin
LGE: Late gadolinium enhancement
NLINV: Nonlinear inverse reconstruction
PSIR: Phase-sensitive inversion recovery FOV, field of view
TE: Echo time
TR: Repetition time
