# Supplementary figures and images for: Targeted endomyocardial biopsy guided by real-time cardiovascular magnetic resonance
Source: J Cardiovasc Magn Reson. 2017 Apr 19;19:45. doi: 10.1186/s12968-017-0357-3 (PMC5395773; doi:10.1186/s12968-017-0357-3)

## Slide 1
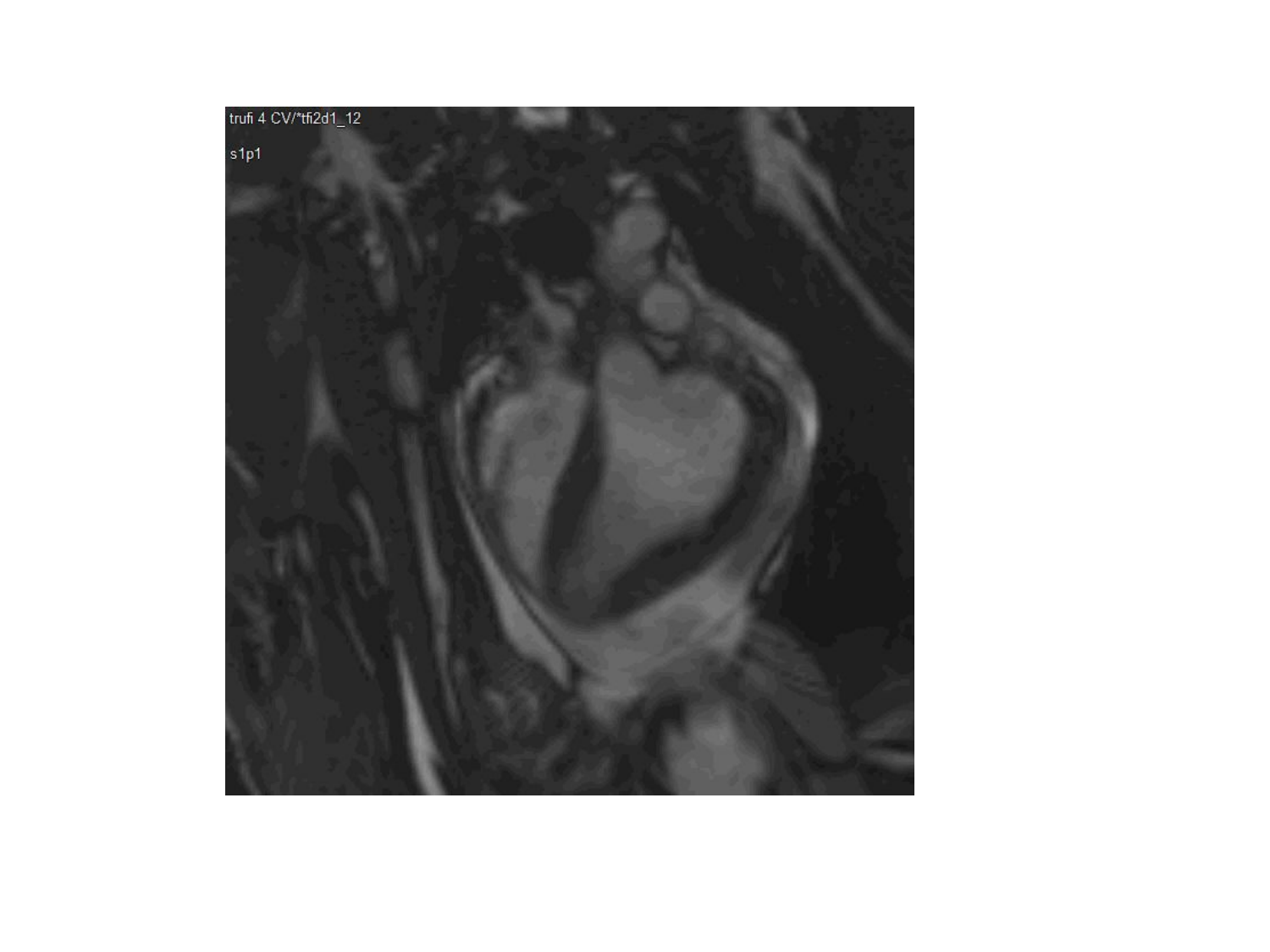

Supplement: Supplementary file 2 — Real-time CMR images seen by the interventionalist during biopsy of a radiofrequency induced lesion similar to the one depicted in Fig. 2. (PPTX 9082 kb) [file 12968_2017_357_MOESM2_ESM.pptx]
